# Dual-Step Solvent Vapor Annealing for Improved Morphology Control in Sequentially Deposited Organic Solar Cells

**DOI:** 10.3390/polym18121435

**Published:** 2026-06-08

**Authors:** Mai Mao, Yuwei Hu, Lidong Liang, Tong Chen, Yitong Ji, Xueyuan Yang, Xiaoxiao You, Wenchao Huang

**Affiliations:** 1 Key State Laboratory of Advanced Technology for Materials Synthesis and Processing, School of Materials Science and Engineering, Wuhan University of Technology, Wuhan 430070, China; 344911@whut.edu.cn (M.M.); 345153@whut.edu.cn (Y.H.); 345192@whut.edu.cn (L.L.); 345107@whut.edu.cn (T.C.); ytji@whut.edu.cn (Y.J.); xueyuan.yang@whut.edu.cn (X.Y.); 2 Chaozhou Branch of Chemistry and Chemical Engineering Guangdong Laboratory, Chaozhou 521000, China

**Keywords:** organic solar cells, sequential deposition, dual-step solvent vapor annealing, morphology control, ultrathin flexible devices

## Abstract

Sequential deposition (SD) processing offers advantages for morphology optimization of active layer and device stability in organic solar cells (OSCs). However, the insufficient solvent resistance of polymer donor layers often leads to uncontrolled interfacial mixing. Herein, we report a dual-step solvent vapor annealing (D-SVA) strategy to address this limitation, without requiring thermal annealing, making it suitable for ultrathin flexible OSCs. Sequential chlorobenzene (CB) and carbon disulfide (CS_2_) vapor treatments enhance the molecular ordering and solvent resistance of the underlying donor layer, while reducing residual solvent and improving interfacial properties. As a result, the rigid SD processed OSCs achieve a power conversion efficiency (PCE) of 19.3%. Moreover, ultrathin flexible devices deliver a PCE of 17.3% with good mechanical stability. This work provides a general and scalable pathway toward high-performance and stable SD organic solar cells.

## 1. Introduction

Organic solar cells (OSCs) have attracted worldwide attention due to their mechanical flexibility, lightweight nature, solution processability, and compatibility with large-area and roll-to-roll fabrication [1,2,3,4,5,6,7,8,9]. In recent years, continuous advances in photovoltaic material design and device engineering have enabled certified power conversion efficiencies (PCE) of single-junction OSCs to exceed 20% [10,11,12,13,14,15,16]. Although the bulk-heterojunction (BHJ) architecture is still widely adopted as the standard active-layer configuration in OSCs, the control of their complex morphology causes persistent challenges. Specifically, the mixing of donor and acceptor materials during BHJ processing often results in poor vertical phase separation [17,18,19]. These unfavorable morphological features restrict efficient charge transport and collection, thereby compromising device performance and operational stability and constituting a critical barrier to the scalable manufacturing of OSCs [20,21].

The sequential deposition (SD) method enables independent and sequential control over the morphology of donor and acceptor components, providing an effective strategy to address these issues [17,19,22,23,24]. Ideally, the active layer prepared by SD can achieve a partially segregated vertical architecture, with the donor enriched near the anode and the acceptor preferentially distributed toward the cathode [25,26,27,28]. Such a partially controlled vertical configuration enlarges donor/acceptor pure-domain sizes and optimizes interfacial morphology, facilitating charge transport and collection. In addition, the SD method demonstrates superior device stability, providing a promising pathway for morphology control in large-area manufacturing of OSCs [29,30,31].

However, solvent from the top-coated acceptor solution often excessively dissolves the underlying donor film, leading to uncontrolled interfacial mixing and the formation of a BHJ-like morphology [32,33,34,35]. To mitigate this issue, several strategies have been proposed, including orthogonal solvent systems [36], multicomponent donor–acceptor material strategies [33], crosslinkers and additives for enhanced donor solvent resistance [34,37,38], and antisolvent treatments for optimized vertical morphology [39]. Nevertheless, these approaches are still limited by strict solvent requirements, increased system complexity, or potential trade-offs in long-term device stability.

Solvent vapor annealing (SVA) has been widely employed to regulate film morphology. In addition, previous studies have reported that SVA can promote crystal growth, indicating increased molecular package [40,41]. However, excessive SVA treatment often results in pronounced domain sizes and the solvent residual in the treated film, which are detrimental to device performance. These limitations highlight the need for a more controllable solvent vapor annealing strategy.

To address these challenges, we propose a simple and broadly applicable dual-step solvent vapor annealing (D-SVA) strategy for optimizing morphology in sequential deposition processing. Specifically, as the first step, CB solvent vapor annealing is applied to the donor film to induce improved crystallization and thus enhancing its resistance to subsequent solvent exposure. Nevertheless, this SVA process is accompanied by the presence of residual solvent within the donor film, which is unfavorable for interfacial morphology. To overcome this, a second solvent vapor annealing step using CS_2_, a solvent with lower boiling point, is subsequently introduced. This dual-step solvent vapor annealing process reduces residual solvent and leads to more ordered molecular packing. As a result, D-SVA-processed devices exhibit an enhanced power conversion efficiency (PCE) of 19.3%, compared with untreated (18.1%) and single SVA-treated (18.6%) devices. Moreover, ultrathin flexible OSCs fabricated with the D-SVA strategy reach a PCE of 17.3%. After 2000 bending cycles, the devices retain over 90% of their initial efficiency. These results demonstrate that the proposed strategy provides an effective approach to improving the performance and operational stability of SD-processed OSCs.

## 2. Materials and Methods

### 2.1. Materials

Ethanol and carbon disulfide (CS_2_, ≥99.0%) were purchased from Aladdin (Shanghai, China). Ethanolamine (≥99.0%) and chlorobenzene (CB, ≥99.8%) were obtained from Sigma-Aldrich (St. Louis, MI, USA). Chloroform (CF, ≥99.0%) was purchased from Yonghua Chemical Co., Ltd. (Suzhou, China). Br-2PACz, PBDB-T-2F (PM6), D18, L8-BO, BTP-eC9, PY-IT, N3, and N, N′-Bis{3-[3-(dimethylamino)propylamino]propyl}perylene-3,4,9,10-tetracarboxylic diimide (PDINN) were sourced from Solarmer Materials Inc. (Beijing, China).

### 2.2. Device Fabrication

Rigid OSCs were fabricated with a conventional glass/ITO/Br-2PACz/active layer/PDINN/Ag architecture. ITO-coated glass substrates were sequentially cleaned by ultrasonication in detergent, deionized water, ethanol, acetone, and isopropyl alcohol, followed by drying under a nitrogen flow and plasma treatment for 15 min. Br-2PACz was used as the hole transport layer (HTL). A 0.3 mg/mL solution in ethanol was sonicated for 30 min, spin-coated onto ITO substrates at 3000 rpm for 45 s, and annealed at 70 °C for 10 min. For sequential deposition (SD) devices, donor and acceptor solutions were prepared in chloroform (CF) as follows. The donor PM6 (6 mg/mL, 3500 rpm) and the acceptor BTP-eC9 (10 mg/mL, 4000 rpm), Y6 (8 mg/mL, 3000 rpm), or PY-IT (8 mg/mL, 3000 rpm) were prepared. Moreover, D18 (4 mg/mL, 2000 rpm) and N3 (8 mg/mL, 3000 rpm) were used for D18/N3 devices.

**Figure 1:**
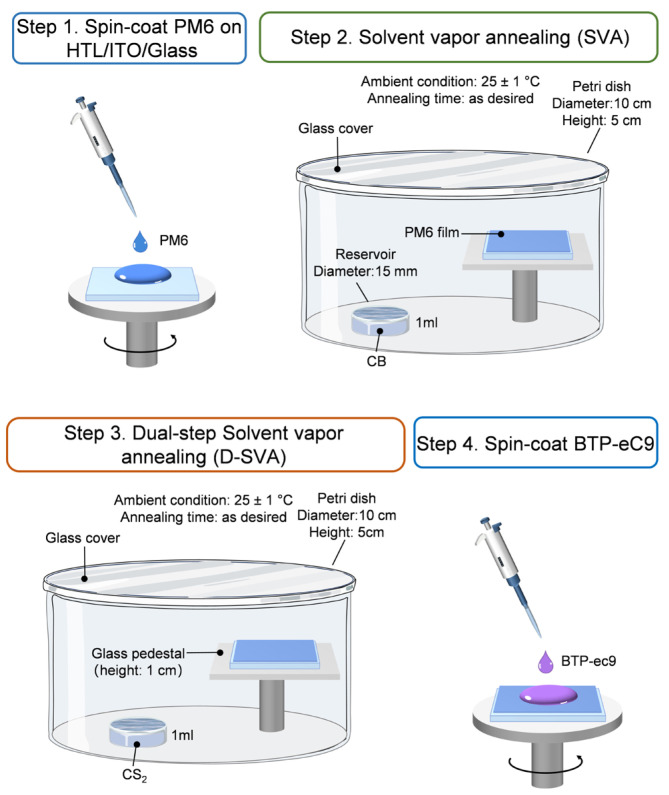
Schematic representation of the dual-step solvent vapor annealing (D-SVA) processes.

The overall fabrication sequence is schematically illustrated in Figure 1. First, a PM6 layer was spin-coated onto HTL/ITO/Glass substrate (Step 1). The film was then subjected to solvent vapor annealing (SVA) with CB (Step 2). For the SVA step, a customized chamber was developed from a standard glass Petri dish (inner diameter: 10 cm). A miniature concave glass reservoir (inner diameter: 15 mm) containing 1 mL of CB was placed at the bottom of the petri dish. The sample was positioned on a glass pedestal (height: 1 cm; length: 3 cm) inside the dish. The petri dish was then covered with its original glass cover, and the sample was annealed under ambient conditions (25 ± 1 °C) for the specified duration. For the dual-step solvent vapor annealing (D-SVA) treatment, the film was then vapor annealed by using CS_2_ (Step 3). Finally, the BTP-eC9 layer was spin-coated on top of the annealed PM6 film (Step 4). It should be noted that the entire procedure was carried out inside a glovebox, where Step 1 and Step 4 correspond to the deposition of the donor and acceptor layers, respectively.

For bulk-heterojunction (BHJ) films, PM6 and BTP-eC9 were blended at a 1:1.2 weight ratio in CF (total concentration of 16.5 mg/mL, 4000 rpm) and spin-coated onto ITO/HTL substrates. PDINN (1.5 mg/mL in methanol, 3000 rpm) was subsequently spin-coated as the electron transport layer, and a 150 nm Ag electrode was thermally evaporated under 2 × 10^−4^ Pa vacuum.

Ultrathin flexible organic solar cells (OSCs) were fabricated with a device structure of parylene/ITO/Br-2PACz/active layer/PDINN/Ag structure. A 3 μm thick parylene film was deposited onto fluoropolymer-coated glass substrates by chemical vapor deposition. A 150 nm indium tin oxide (ITO) layer was then sputtered and patterned through a shadow mask, resulting in a sheet resistance of approximately 40 Ω sq^−1^. Unless otherwise specified, all subsequent fabrication procedures were identical to those used for rigid devices.

### 2.3. Characterization

The absorption spectra were recorded using a UV–Vis spectrophotometer (UV-1900i, Shimadzu, Japan). Thermal desorption/pyrolysis-gas chromatography–mass spectrometry measurements were performed using a TurboMatrix ATD 350 (Waltham, MA, USA) coupled with an Atomx P&T system (West Jefferson, OH, USA) and an Agilent 7890B-5977B GC-MS (Santa Clara, CA, USA). Samples were desorbed at 60 °C, and the analysis was conducted over a 2–12 min time range.

Device *J*-*V* characteristics were measured under simulated AM 1.5G solar illumination (100 mW cm^−2^) using a solar simulator (SS-X50, Enlitech, Shanghai, China), with an effective device area of 0.04 cm^2^ defined by a metal shadow mask. External quantum efficiency (EQE) spectra were obtained using a solar-cell spectral response measurement system (QE-R, Enlitech, Shanghai, China), with a calibrated standard Si solar cell for light intensity calibration. Transient photocurrent (TPC) and transient photovoltage (TPV) measurements were performed using a transient measurement system (LST-TPC, Shanghai Jinzhu Technology Co., Ltd., Shanghai, China). Atomic force microscopy (AFM) measurements were performed using a Cypher ES system (Asylum Research, Oxford Instruments, Oxfordshire, UK). Grazing-incidence wide-angle X-ray scattering (GIWAXS) measurements were carried out on a Xeuss 3.0 SAXS/WAXS laboratory system (Xenocs, Grenoble, France) equipped with a Cu Kα X-ray source (8.05 keV, λ = 1.54 Å) and a Pilatus 100K detector, with a sample-to-detector distance of 80 mm and an incident angle fixed at 0.18°.

Water contact angles on the active layer surfaces were measured using a video-optical contact angle meter (ThetaLite100, Biolin Scientific, Gothenburg, Sweden). X-ray photoelectron spectroscopy (XPS) measurements were conducted using a Thermo Scientific Escalab 250Xi spectrometer (Thermo Fisher Scientific, Waltham, MA, USA).

The positive and negative electrodes of the flexible devices were connected to the measurement system via external wiring for electrical characterization. Bending and cyclic compression–stretching tests were performed using an electronic mechanical testing system (PR-BDM4-100V, Purui Materials Technology Co., Ltd., Shenzhen, China) capable of precisely controlling the bending and compression conditions. The electrical performance during the mechanical stability tests was monitored using a Keithley 2450 source-measure unit (Keithley Instruments, Cleveland, OH, USA).

## 3. Results

### 3.1. Film Properties

**Figure 2:**
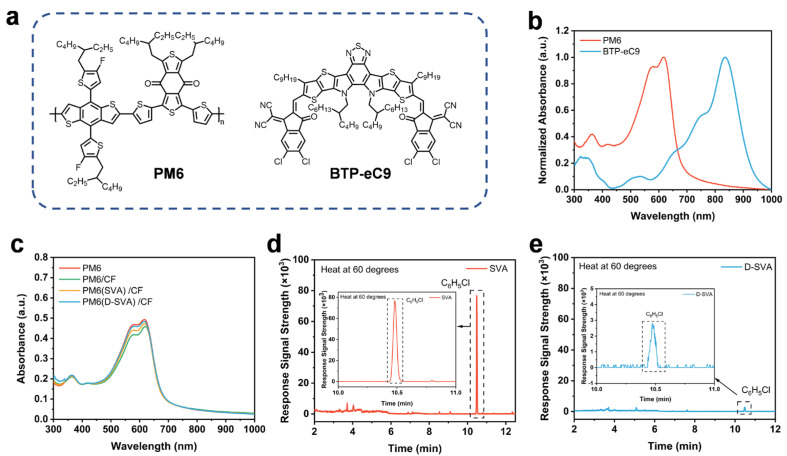
(**a**) Molecular structures of PM6 and BTP-eC9. (**b**) Normalized UV-Vis absorption spectra of PM6 and BTP-eC9 thin films. (**c**) UV-Vis absorption spectra of PM6 donor films with different treatments before and after chloroform rinsing. (**d**,**e**) Thermal desorption/pyrolysis–gas chromatography–mass spectrometry (TD/P&T-GC-MS) analysis of residual solvent in PM6 films treated with solvent vapor annealing (SVA) and dual-step solvent vapor annealing (D-SVA), respectively.

In this work, PM6 and BTP-eC9 are employed as the donor and acceptor, respectively, with their molecular structures shown in Figure 2a. PM6 contains fluorine (F), while BTP-eC9 incorporates chlorine (Cl), enabling subsequent elemental analysis. The energy level alignment of the two materials is presented in Figure S1. As shown in Figure 2b, the normalized UV-Vis absorption spectra of PM6 and BTP-eC9 exhibit good spectral complementarity. To evaluate the influence of different processing strategies on the solvent resistance of the donor layer, PM6 films subjected to no treatment, solvent vapor annealing (SVA) and dual-step solvent vapor annealing (D-SVA) are systematically investigated. Figure 2c compares the UV-Vis absorption spectra of PM6 films before and after rinsing with chloroform (CF). The untreated PM6 film exhibits a clear decrease in absorption intensity after CF rinsing, indicating notable film damage during acceptor deposition. In contrast, SVA-treated PM6 films show improved solvent resistance, while a reduction in absorption intensity is still observed. This behavior is consistent with the presence of residual chlorobenzene (CB) from the first SVA step in the film. Notably, PM6 films subjected to the D-SVA treatment maintain absorption characteristics comparable to those of the pristine film, demonstrating enhanced solvent resistance. Similarly, UV-Vis absorption spectra of PM6/BTP-eC9 bilayer films also indicate that the D-SVA treatment improves solvent resistance (Figure S2). To further evaluate the residual solvent content throughout the entire donor film, thermal desorption/pyrolysis–gas chromatography–mass spectrometry (TD/P&T-GC-MS) measurements are performed (Figure 2d,e). The results show that CB is detectable in films treated only with the first SVA step, whereas the subsequent introduction of a high-vapor-pressure CS_2_ vapor annealing step results in a reduced residual solvent signal. In addition, no CS_2_ signal is observed under the standard processing, consistent with the detection limit confirmed by a control experiment (Figure S3), where CS_2_ is detectable.

### 3.2. Device Performance and Physics

**Figure 3:**
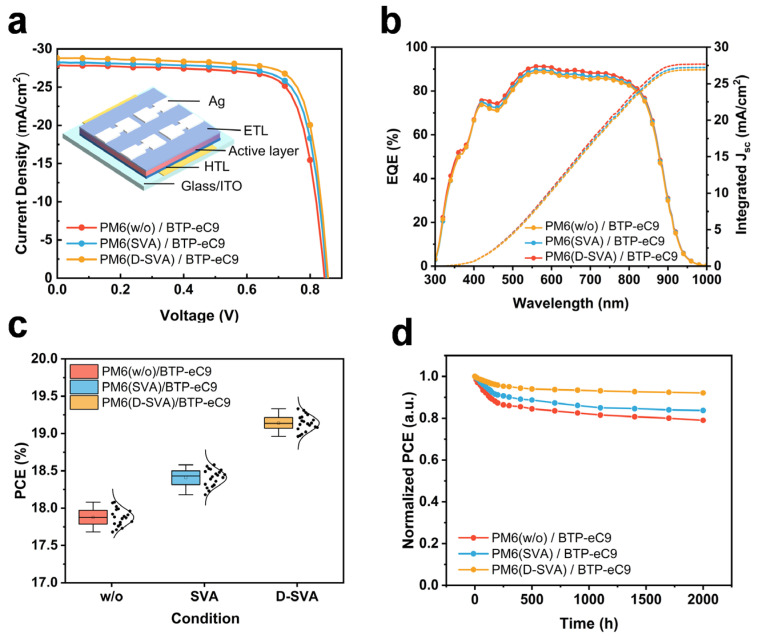
Photovoltaic performance of rigid organic solar cells (OSCs) with different solvent annealing treatments. (**a**) *J-V* curves of rigid OSCs. (**b**) EQE spectra of rigid OSCs. (**c**) Statistics of power conversion efficiency (PCE) counts for 20 rigid OSCs. (**d**) Storage stability of rigid OSCs under an N_2_ atmosphere.

The device architecture consists of indium tin oxide (ITO)/Br-2PACz/PM6/BTP-eC9/PDINN/Ag. The optimization of the dual-step solvent vapor annealing (D-SVA) process is shown in Figures S4 and S5, with detailed photovoltaic parameters summarized in Table S1. The best device performance is achieved with a 5 min solvent annealing treatment using the first solvent, followed by a 40 s exposure to the second solvent. Figure 3a presents the current density-voltage (*J-V*) characteristics of devices without any treatment (w/o), with chlorobenzene solvent vapor annealing (SVA), and with dual-step solvent vapor annealing (D-SVA). The corresponding photovoltaic parameters are listed in Table 1. Devices without treatment exhibit a power conversion efficiency (PCE) of 17.8%, with an open-circuit voltage (*V_OC_*) of 0.846 V, a short-circuit current density (*J_SC_*) of 27.9 mA cm^−2^, and a fill factor (FF) of 76.8%. After SVA treatment, the PCE increases to 18.6%, along with *V_OC_* of 0.852 V, *J_SC_* of 28.2 mA cm^−2^, and FF of 77.4%. With the implementation of the D-SVA strategy, the PCE reaches 19.3%, and the corresponding *V_OC_*, *J_SC_*, and FF are 0.857 V, 28.7 mA cm^−2^, and 78.3%, respectively. Figure 3b shows the external quantum efficiency (EQE) spectra of devices processed under different conditions. The current densities calculated from EQE integration show deviations of less than 5% from those obtained from the *J-V* measurements, indicating consistency between the two measurement methods. A comparative summary of representative PM6-based rigid sequentially deposited OSCs is provided in Figure S6 and Table S2, showing that our devices perform similarly to prior reports without relying on complex processing protocols. 

To evaluate the device reproducibility of the D-SVA strategy, statistical analysis of the PCEs from 20 devices is conducted (Figure 3c), showing that D-SVA-treated devices exhibit an enhanced reproducibility. Corresponding box plots of *V_OC_*, *J_SC_*, and FF are provided in Figure S7. Furthermore, after storage in a nitrogen-filled glovebox for 2000 h (Figure 3d), the D-SVA-treated devices exhibit the smallest decrease in efficiency. In contrast, devices treated with SVA and those without treatment display larger efficiency losses during the same storage period. Moreover, the general applicability of the D-SVA strategy is evaluated across multiple material systems beyond PM6/BTP-eC9, including D18/N3, PM6/Y6, and PM6/PY-IT (Figures S8–S10, Table S3). Performance improvements are observed in all tested systems. Although the optimal solvent vapor exposure time should be adjusted for each donor materials, the D-SVA strategy is broadly applicable across multiple material systems.

**Figure 4:**
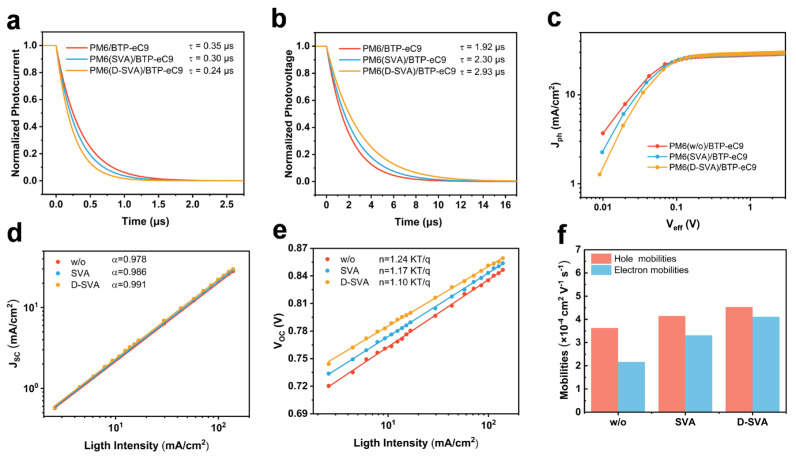
Device physics of rigid organic solar cells (OSCs). (**a**) Transient photocurrent (TPC) measurement of OSCs. (**b**) Transient photovoltage (TPV) measurement of OSCs. (**c**) Photocurrent density as a function of effective voltage (*J_ph_-V_eff_*). (**d**) Dependence of *J_SC_* on light intensity. (**e**) Dependence of *V_OC_* on light intensity. (**f**) Comparison of hole and electron charge carrier mobility.

To examine the charge carrier dynamics associated with the simultaneous enhancement in *J_SC_* and FF upon D-SVA treatment, transient photocurrent (TPC) and transient photovoltage (TPV) measurements are carried out (Figure 4a,b) [42]. As shown in Figure 4a, the TPC decay profiles reveal that devices treated with D-SVA exhibit the shortest charge extraction time of 0.24 μs, compared with 0.35 μs and 0.30 μs for untreated and SVA-treated devices, respectively. The reduced charge extraction time is consistent with the higher *J_SC_* values observed in D-SVA-treated devices. Complementary insights into charge recombination processes are obtained from TPV decay measurements (Figure 4b). The carrier recombination lifetime of D-SVA-treated devices reaches 2.93 μs, which is longer than that of untreated devices (1.92 μs) and SVA-treated devices (2.30 μs). This extended recombination lifetime corresponds to the improved FF observed in the D-SVA-treated devices.

Additionally, as a complementary analysis, the photocurrent density (*J_ph_*) as a function of effective voltage (*V_eff_*) is measured to further characterize the charge extraction behavior of the devices (Figure 4c and Table S4) [43]. When *V_eff_* exceeds 2.0 V, the *J_ph_*-*V_eff_* curves reach saturation, corresponding to the saturated photocurrent density (*J_sat_*). Based on the *J_ph_*-*V_eff_* characteristics, the calculated exciton dissociation efficiency (*η_diss_*) is determined to be 98.2% for the D-SVA-treated device, compared to 97.6% for the SVA-treated device and 96.9% for the untreated device. 

The dependence of *J_SC_* and *V_OC_* on light intensities (*P_light_*) is studied to investigate the impact of D-SVA treatment on charge transport and recombination in OSCs (Figure 4d,e). The D-SVA-treated devices exhibit an α value of 0.991, compared with 0.978 for devices without treatment and 0.986 for SVA-treated devices. An α value closer to unity is generally associated with reduced bimolecular recombination [44], and the observed trend is consistent with the device performance. 

The slope of the *V_OC_* versus *P_light_* plot theoretically ranges between kT/q and 2 kT/q. A slope close to kT/q suggests dominant bimolecular recombination, while a slope near 2 kT/q implies trap-assisted recombination [45]. As shown in Figure 4e, the D-SVA-treated devices exhibit a slope of 1.10 kT/q, which is lower than that of untreated (1.24 kT/q) and SVA-treated devices (1.17 kT/q). This reduced slope suggests a lower contribution from trap-assisted recombination in the D-SVA-treated devices. In addition, the the hole mobility (*μ_h_*) increases from 3.63 × 10^−4^ to 4.53 × 10^−4^ cm^2^ V^−1^ s^−1^ in the film after D-SVA treatment (Figure 4f).Consequently, the mobility ratio (*μ_h_/μ_e_*) changes from 1.67 to 1.10 (Table S5), indicating a more balanced charge transport behavior. Collectively, these results indicate improved charge dynamics and recombination behavior in D-SVA-treated devices, consistent with enhanced device performance.

### 3.3. Morphological Characterization

**Figure 5:**
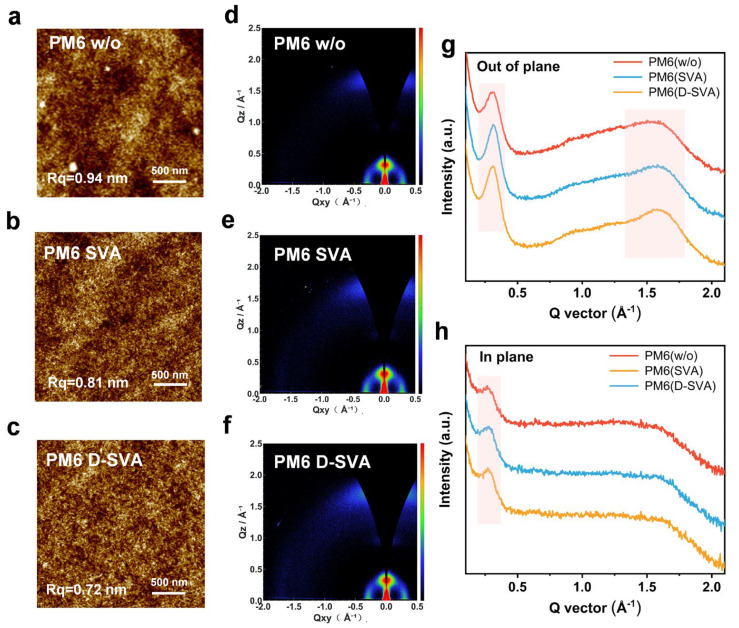
Morphological and molecular packing characterization of PM6 donor films. Atomic force microscopy (AFM) images of PM6 films (**a**) without treatment (w/o), (**b**) after solvent vapor annealing (SVA), and (**c**) after dual-step solvent vapor annealing (D-SVA). 2D GIWAXS patterns of donor materials (**d**) w/o, (**e**) SVA-treated, and (**f**) D-SVA-treated. Corresponding GIWAXS 1D profiles along (**g**) out-of-plane and (**h**) in-plane directions.

The morphology and crystallization behavior of the donor films optimized by the dual-step solvent vapor annealing (D-SVA) strategy is investigated using atomic force microscopy (AFM) and grazing-incidence wide-angle X-ray scattering (GIWAXS), as shown in Figure 5. The AFM images reveal that the untreated PM6 film exhibits higher surface roughness (root-mean-square roughness, Rq = 0.94 nm), as shown in Figure 5a. After solvent vapor annealing (SVA), these surface features are reduced (Figure 5b), and the roughness decreases to Rq = 0.81 nm. With the application of the D-SVA treatment (Figure 5c), the surface roughness is further reduced to Rq = 0.72 nm. Meanwhile, as shown in Figure S11, AFM images (200 nm scan size) indicates the fibrous structures in the D-SVA-treated film. These morphological features are consistent with increased molecular ordering in the PM6 film. Notably, a comparison of the bilayer film morphologies (Figure S12) shows that the surface roughness of bilayer films treated with single-step SVA (Rq = 2.25 nm) is similar to that of untreated bilayer films (Rq = 2.27 nm). In contrast, bilayer films processed using the D-SVA strategy exhibit a lower surface roughness (Rq = 1.37 nm).

Figure 5d–h show 2D GIWAXS patterns of PM6 films subjected to different processing conditions, together with the corresponding 1D line cuts. The extracted crystallographic parameters are summarized in Table S6. In the out-of-plane (OOP) direction, the untreated PM6 film exhibits a lamellar (100) diffraction peak at q_z_ = 0.327 Å^−1^ (d = 19.21 Å), with a crystalline coherence length (CCL) of 53.3 Å. After single-solvent vapor annealing, the peak shifts to q_z_ = 0.332 Å^−1^ (d = 18.93 Å), accompanied by an increased CCL of 56.0 Å. With the implementation of the dual-step solvent vapor annealing treatment, the (100) peak position further shifts to q_z_ = 0.341 Å^−1^ (d = 18.43 Å), while the CCL increases to 58.9 Å, corresponding to a reduced lamellar spacing and enhanced molecular ordering. 

Meanwhile, the CCL associated with the OOP (010) π-π stacking peak also increases, reflecting improved π-π stacking coherence. Similar trends are observed in the in-plane (IP) direction, where the CCL of the (100) diffraction peak increases from 67.3 Å in the untreated film to 73.4 Å after D-SVA treatment. For comparison, 2D GIWAXS patterns and corresponding 1D line cuts of BTP-eC9 single-component films and PM6/BTP-eC9 bilayer active layers are provided in Figures S13 and S14, with the extracted crystallographic parameters summarized in Tables S7 and S8. The GIWAXS results of the bilayer active layers exhibit microstructural evolution trends similar to those observed in the donor films, indicating that the crystallographic features introduced by the D-SVA treatment are retained in the bilayer configuration. This behavior is consistent with a solvent-vapor-induced recrystallization process, where CS_2_ facilitates molecular rearrangement upon rapid evaporation [46].

### 3.4. Surface Composition

**Figure 6:**
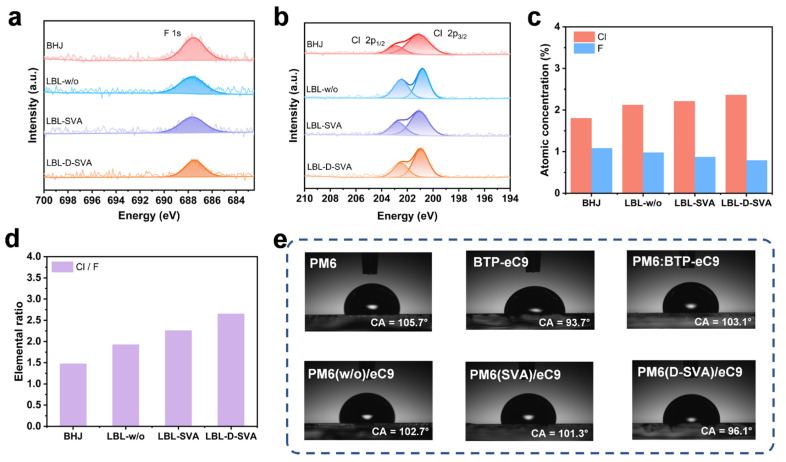
Surface composition analysis of active layers. X-ray photoelectron spectroscopy (XPS) analysis of mixed BHJ and SD films with different treatments: (**a**) F 1s and (**b**) Cl 2p core-level spectra. (**c**) Atomic percentages of F and Cl near the cathode-side surface. (**d**) Cl/F atomic ratio. (**e**) Water contact angles of different films.

X-ray photoelectron spectroscopy (XPS) elemental analysis, combined with water contact angle measurements, is employed to probe the elemental ratios and vertical distribution at the top-side of the active layer. The corresponding atomic percentages are summarized in Table S9. Figure 6a and Figure 6b show the characteristic F (from PM6) and Cl (from BTP-eC9) spectra under different processing conditions, while other elemental spectra are provided in Figure S15. As shown in Figure 6c,d, the D-SVA-treated films exhibit an increased Cl content and a higher Cl/F atomic ratio at the surface. These results indicate that D-SVA suppresses donor dissolution and downward interdiffusion, leading to preferential acceptor enrichment at the cathode-side surface. Consistently, water contact angle measurements (Figure 6e) show that the D-SVA-treated bilayer film exhibits a contact angle closer to that of pristine BTP-eC9, confirming the formation of a more acceptor-like surface. To further probe the vertical compositional distribution, depth-profiled XPS measurements were conducted (Figure S16). As the etching time increases, the Cl signal (from BTP-eC9) gradually decreases, accompanied by a corresponding increase in the F signal (from PM6), revealing a clear compositional gradient from an acceptor-enriched surface toward a mixed region. This observation provides direct evidence for the vertical phase separation induced by the D-SVA treatment.

### 3.5. Ultrathin Flexible OSCs

**Figure 7:**
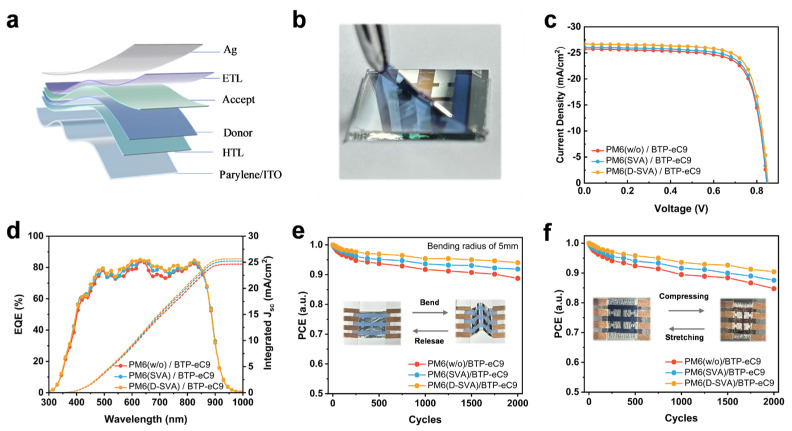
Photovoltaic performance and mechanical stability of ultrathin flexible OSCs. (**a**) Device structure of ultrathin flexible OSCs. (**b**) Photos of ultrathin flexible OSCs. (**c**) *J-V* characteristics of ultrathin flexible OSCs. (**d**) EQE spectra of ultrathin flexible OSCs. (**e**) Normalized efficiency as a function of bending cycles. (**f**) Normalized efficiency as a function of the compression–stretching cycles.

Ultrathin flexible OSCs with a device structure of parylene/ITO/HTL/PM6/BTP-eC9/ETL/Ag are fabricated following our previously reported procedures [47]. The device structure and photos of ultrathin flexible OSCs are shown in Figure 7a and Figure 7b, respectively. The *J-V* characteristics and schematic device structure are shown in Figure 7c, with the corresponding photovoltaic parameters summarized in Table S10. The D-SVA-treated ultrathin flexible OSCs achieve a PCE of 17.3%, outperforming devices processed with SVA (16.7%) and those without treatment (16.3%). Consistently, an enhanced EQE across the visible spectral range is observed for the D-SVA-treated devices (Figure 7d). A comparative overview of reported ultrathin flexible OSCs is provided in Figure S17 and Table S11. The D-SVA-treated devices in this work achieve one of the highest reported efficiencies among comparable ultrathin flexible OSCs, demonstrating the effectiveness of the strategy in achieving high performance under mechanically compliant conditions.

The mechanical stability of the flexible devices is further evaluated by cyclic bending and compression–stretching tests. After 2000 bending cycles, the D-SVA-treated devices retain 94.0% of their initial efficiency, compared with 91.8% for SVA-treated and 88.7% for untreated devices (Figure 7e). As shown in Figure S18, ultrathin flexible OSCs are transferred to an elastomeric material (3 m VHB) that is pre-stretched by 200%. Under 50% compressive strain, the D-SVA-treated devices maintain 90.4% of the initial efficiency after 1000 compression–stretching cycles, significantly higher than those of untreated (84.7%) and SVA-treated (87.5%) devices (Figure 7f). These results demonstrate that the D-SVA strategy simultaneously enhances efficiency and mechanical durability in ultrathin flexible OSCs, highlighting its potential for flexible and wearable photovoltaic applications.

## 4. Conclusions

In this work, we present a simple and broadly applicable dual-step solvent vapor annealing (D-SVA) strategy for improving donor solvent resistance and regulating vertical morphology in sequential deposition (SD) organic solar cells. Using the PM6/BTP-eC9 system as a model, sequential chlorobenzene (CB) and high-vapor-pressure carbon disulfide (CS_2_) vapor annealing induce donor crystallization and reduce residual solvent, enabling optimized interfacial morphology and component distribution. Correspondingly, enhanced charge transport and reduced recombination signatures are observed, resulting in a power conversion efficiency of 19.3% in SD-processed organic solar cells. Moreover, the D-SVA strategy applies to multiple donor–acceptor systems and can be extended to ultrathin flexible devices, which exhibit improved mechanical durability and operational stability. 

## Figures and Tables

**Table 1:** Photovoltaic parameters of SD devices based on various donor–acceptor systems under AM 1.5 G illumination at 100 mW cm^−2^.

Treatment	*V_OC_* (V)	*J_SC_* (mA/cm^2^)	*J_SC_* (EQE) (mA/cm^2^)	FF (%)	PCE (%)
w/o	0.846 (0.844 ± 0.002)	27.9 (27.6 ± 0.3)	26.9	76.8 (76.1 ± 0.7)	18.1 (17.8 ± 0.3)
SVA	0.852 (0.849 ± 0.003)	28.2 (27.9 ± 0.3)	27.2	77.4 (76.9 ± 0.5)	18.6 (18.4 ± 0.2)
D-SVA	0.857 (0.855 ± 0.002)	28.8 (28.6 ± 0.2)	27.7	78.3 (78.1 ± 0.2)	19.3 (19.1 ± 0.2)

## Data Availability

The data supporting the findings of this study are available within the article and its Supplementary Information.

## Supplementary Materials

The following supporting information can be downloaded at: https://www.mdpi.com/article/10.3390/polym18121435/s1, Figure S1: Energy level diagram of the materials; Figure S2: UV-Vis absorbance spectra of PM6/BTP-eC9 bilayer films with different treatments; Figure S3: Thermal desorption/pyrolysis–gas chromatography–mass spectrometry (TD/P&T-GC-MS) analysis of residual solvent in PM6 films treated with the reversed solvent vapor annealing sequence: first CS_2_ SVA, then CB SVA; Figure S4: *J-V* curves of rigid devices based on different treatment times. (a) CB 0 min, (b) CB 1 min, (c) CB 3 min, (d) CB 5 min, (e) CB 7 min, (f) CB 10 min; Figure S5: EQE spectra of rigid devices based on different treatment times. (a) CB 0 min, (b) CB 1 min, (c) CB 3 min, (d) CB 5 min, (e) CB 7 min, (f) CB 10 min; Figure S6: Summary of power conversion efficiencies (PCE) of sequentially deposited rigid organic solar cells based on PM6 donor under different strategies; Figure S7: Statistics of the (a) *V_OC_*, (b) *J_SC_*, and (c) FF of OSCs with different treatments based on 20 devices; Figure S8: Chemical structures of different donors and acceptors; Figure S9: UV-Vis absorbance spectra of different donors and acceptors; Figure S10: *J-V* curves and EQE spectra of rigid devices with different active layers. (a, b) PM6/Y6, (c, d) D18/N3, (e, f) PM6/PY-IT; Figure S11: Atomic force microscopy (AFM) images of PM6 donor films. (a) without treatment (w/o), (b) after solvent vapor annealing (SVA), and (c) after dual-step solvent vapor annealing (D-SVA). Scale bar: 200 nm; Figure S12: AFM images of bilayer active-layer films based on PM6 films subjected to different treatment methods. (a) without treatment (w/o), (b) solvent vapor annealing (SVA), (c) dual-step solvent vapor annealing (D-SVA); Figure S13: The 2D GIWAXS scattering patterns of different active layers. (a) BTP-eC9, (b) PM6 (w/o)/BTP-eC9, (c) PM6 (SVA)/BTP-eC9, (d) PM6 (D-SVA)/BTP-eC9; Figure S14: Line scattering profiles cut from the 2D GIWAXS patterns of different active layers in out-of-plane (OOP) and in-plane (IP) directions. (a) BTP-eC9, (b) PM6 (w/o)/BTP-eC9, (c) PM6 (SVA)/BTP-eC9, (d) PM6 (D-SVA)/BTP-eC9; Figure S15: XPS spectra of different elements in active layers. (a) C, (b) N, (c) O, (d) S; Figure S16: Depth-profiled XPS (DP-XPS) analysis of Cl and F elements in the film. (a) Cl 2p spectra at different etching times, (b) F 1s spectra at different etching times, (c) Cl/F elemental ratio and Cl, F atomic concentration versus etching time; Figure S17: Summary of power conversion efficiencies (PCE) of ultrathin flexible organic solar cells; Figure S18: Photographs of ultrathin flexible organic solar cells at different compression levels. Table S1: Photovoltaic parameters of PM6/BTP-eC9 devices for different optimization times; Table S2: The statistical data of sequentially deposited rigid organic solar cells based on PM6 donor under different strategies; Table S3: Summary of rigid OSCs photovoltaic parameters for different active layer systems; Table S4: *J_ph_*, *J_sat_*, and *J_ph_*/*J_sat_* values of different processed devices; Table S5: Electron and hole mobilities and mobility ratios for different treatments; Table S6: Analytical data of GIWAXS results for PM6 films under different treatment methods; Table S7: Analytical data of GIWAXS results for BTP-eC9 films; Table S8: Analytical data of GIWAXS results for bilayer active-layer films under different treatment methods; Table S9: Atomic concentrations of active layers obtained from XPS under different treatment methods; Table S10: Summary of ultrathin flexible OSCs photovoltaic parameters based on different treatments; Table S11: The statistical data for ultrathin flexible organic solar cells. References [48,49,50,51,52,53,54,55,56,57,58,59,60,61,62,63,64,65] are cited in the Supplementary Materials.
