# Supplementary material for: Dual-Step Solvent Vapor Annealing for Improved Morphology Control in Sequentially Deposited Organic Solar Cells
Source: Polymers (Basel). 2026 Jun 8;18(12):1435. doi: 10.3390/polym18121435 (PMC13306702; doi:10.3390/polym18121435)
Supplement: Supplementary file 1 [file polymers-18-01435-s001.zip › polymers-4293671-supplementary.pdf]

Supporting information

Dual-Step Solvent Vapor Annealing for Improved Morphology Control in Sequentially Deposited Organic Solar Cells

Mai Mao[1], Yuwei Hu[1], Lidong Liang[1], Tong Chen[1], Yitong Ji[1], Xueyuan Yang[1,2], Xiaoxiao You[1*] and Wenchao Huang[1*]

[1]Key State Laboratory of Advanced Technology for Materials Synthesis and Processing, School of Materials Science and Engineering, Wuhan University of Technology, 430070, Wuhan, China

[2]Chaozhou Branch of Chemistry and Chemical Engineering Guangdong Laboratory, Chaozhou, 521000, China

[*]Correspondence: xiaoxiaoyou@whut.edu.cn (X.Y.); wenchao.huang@whut.edu.cn (W.H.)

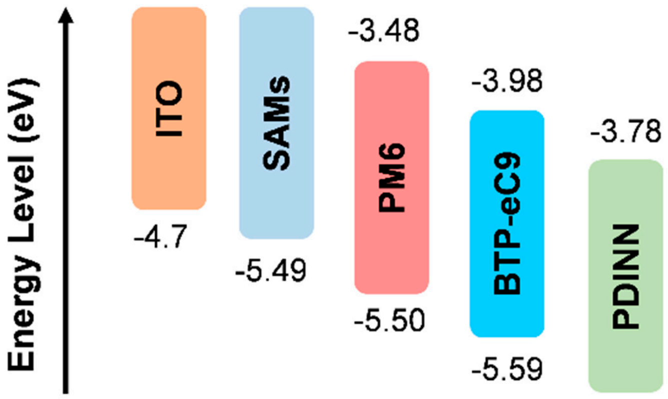

Figure S1. Energy level diagram of the materials.

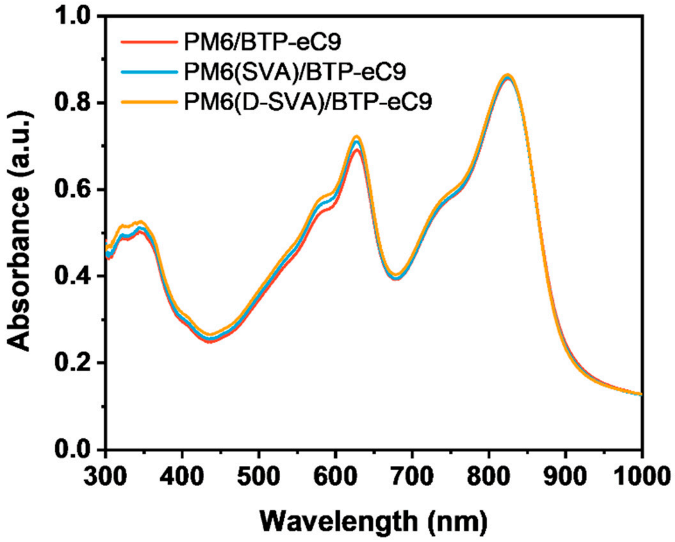

Figure S2. UV-Vis absorbance spectra of PM6/BTP-eC9 bilayer films with different treatments.

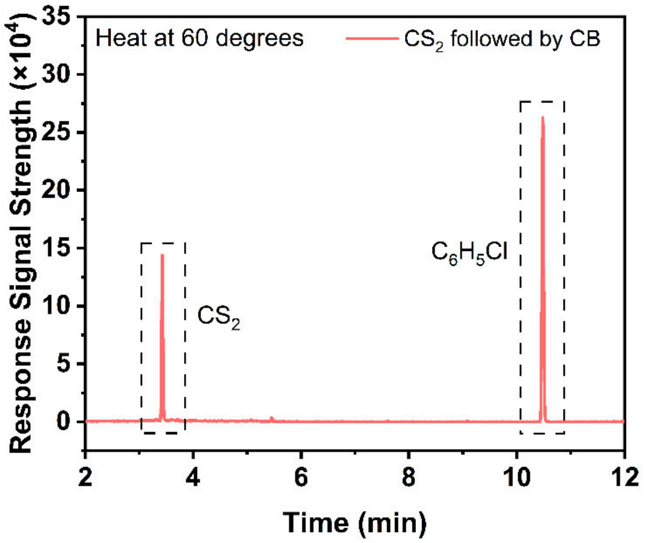

Figure S3. Thermal desorption/pyrolysis–gas chromatography–mass spectrometry (TD/P&T-GC-MS) analysis of residual solvent in PM6 films treated with the reversed solvent vapor annealing sequence: first CS_2 SVA, then CB SVA.

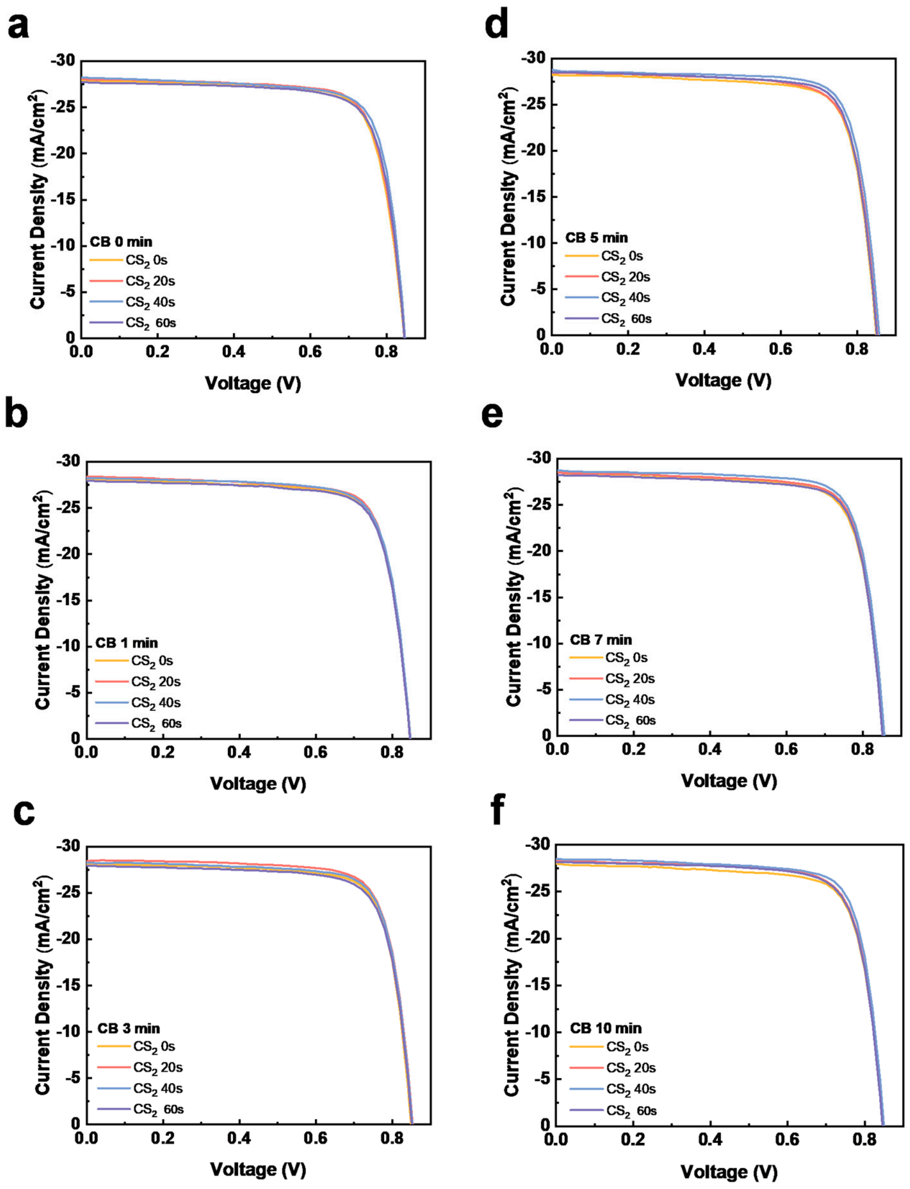

Figure S4. *J-V* curves of rigid devices based on different treatment times. (a) CB 0 min, (b) CB 1 min, (c) CB 3 min, (d) CB 5 min, (e) CB 7 min, (f) CB 10 min.

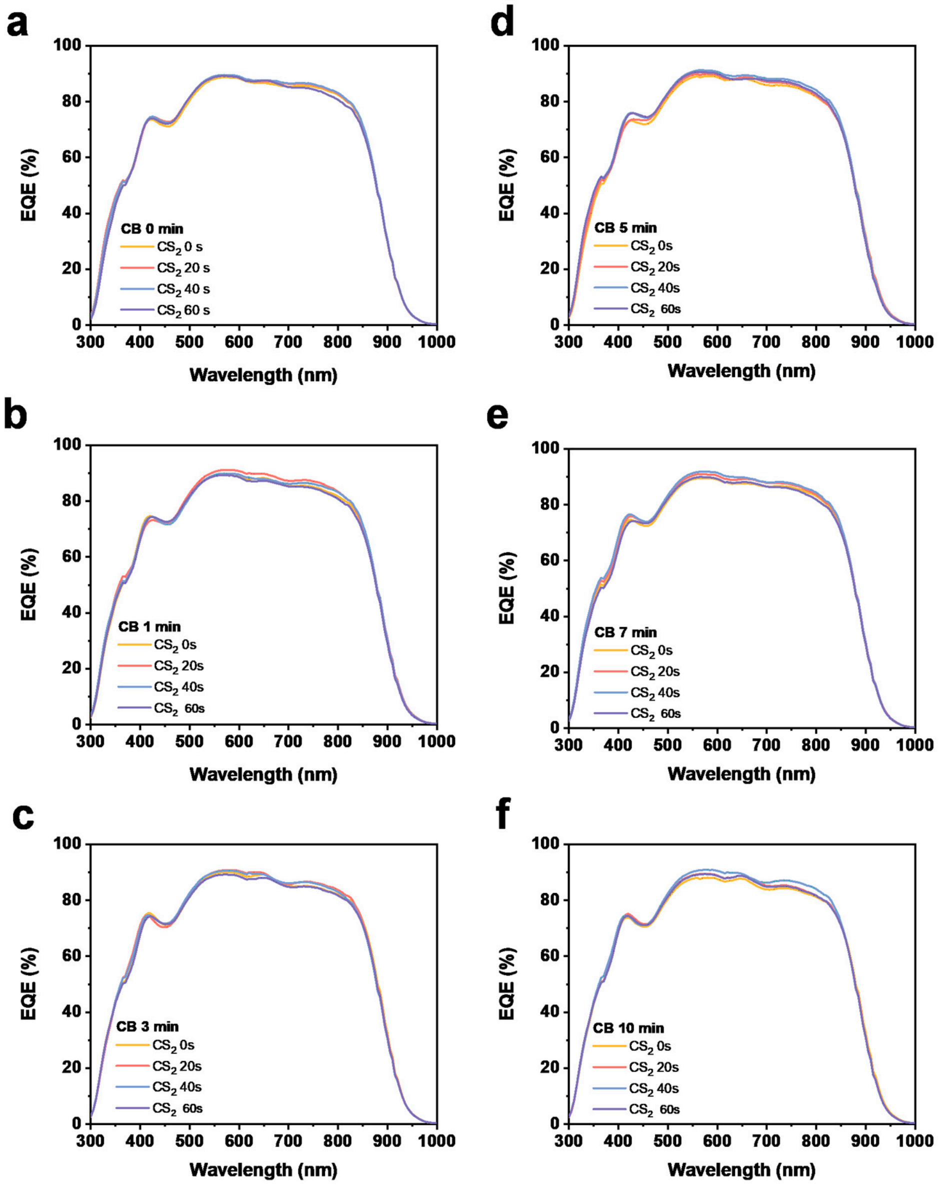

Figure S5. EQE spectra of rigid devices based on different treatment times. (a) CB 0 min, (b) CB 1 min, (c) CB 3 min, (d) CB 5 min, (e) CB 7 min, (f) CB 10 min.

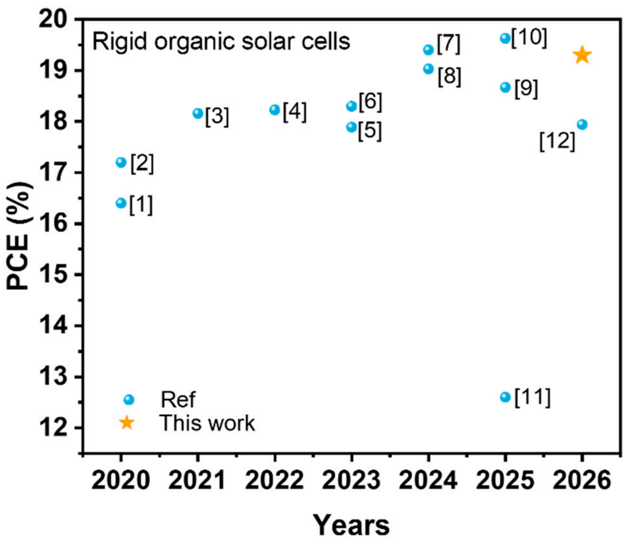

Figure S6. Summary of power conversion efficiencies (PCE) of sequentially deposited rigid organic solar cells based on PM6 donor under different strategies.

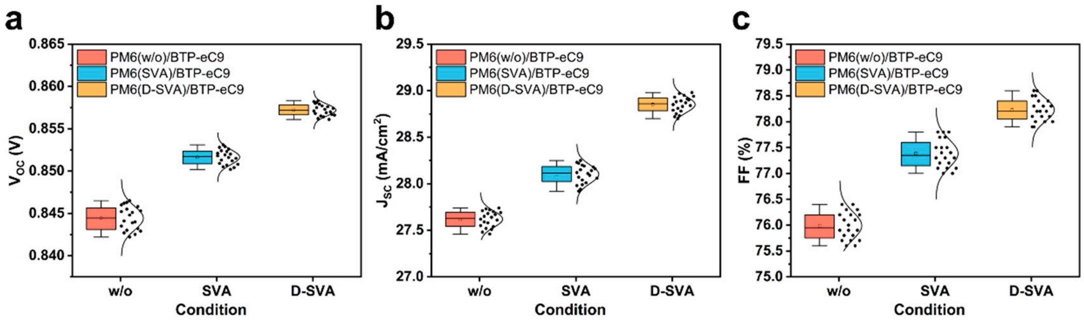

Figure S7. Statistics of the (a) V_{OC}, (b) J_{SC}, and (c) FF of OSCs with different treatments based on 20 devices.

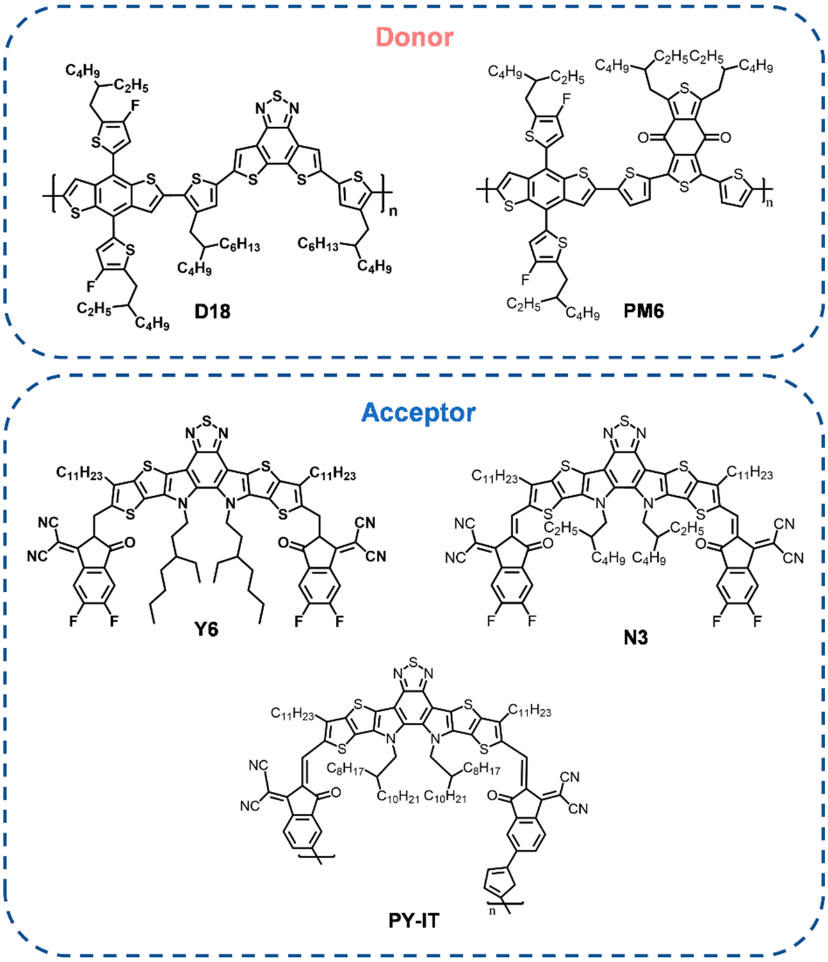

Figure S8. Chemical structures of different donors and acceptors.

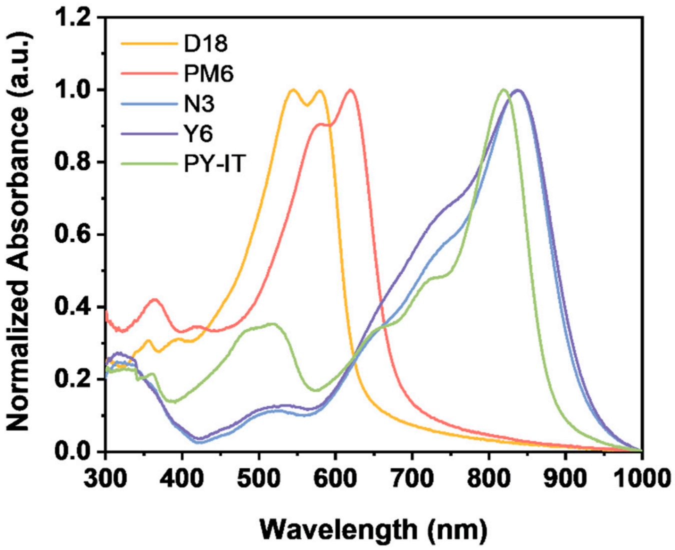

Figure S9. UV-Vis absorbance spectra of different donors and acceptors.

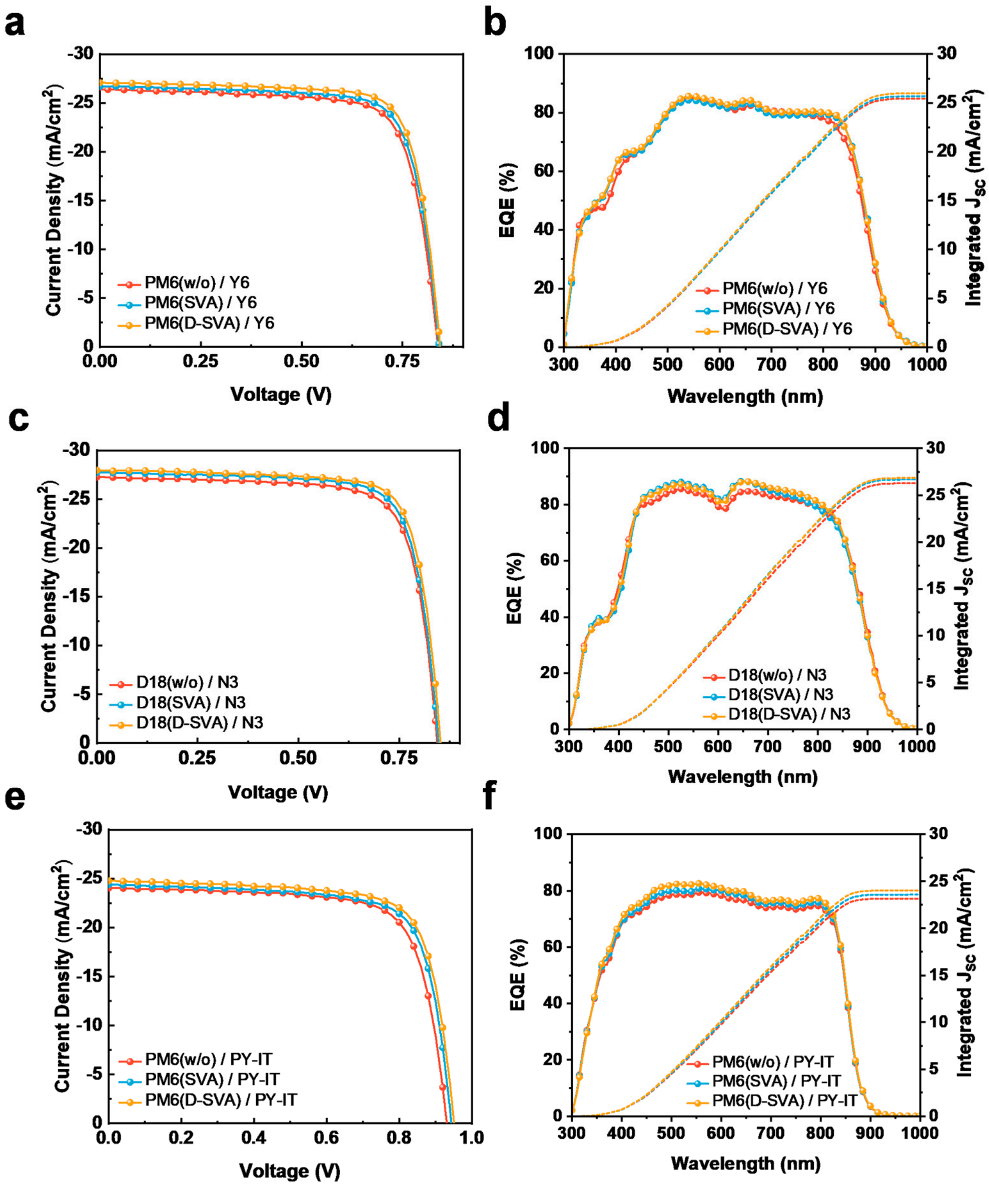

Figure S10. *J*-*V* curves and EQE spectra of rigid devices with different active layers. (a, b) PM6/Y6, (c, d) D18/N3, (e, f) PM6/PY-IT.

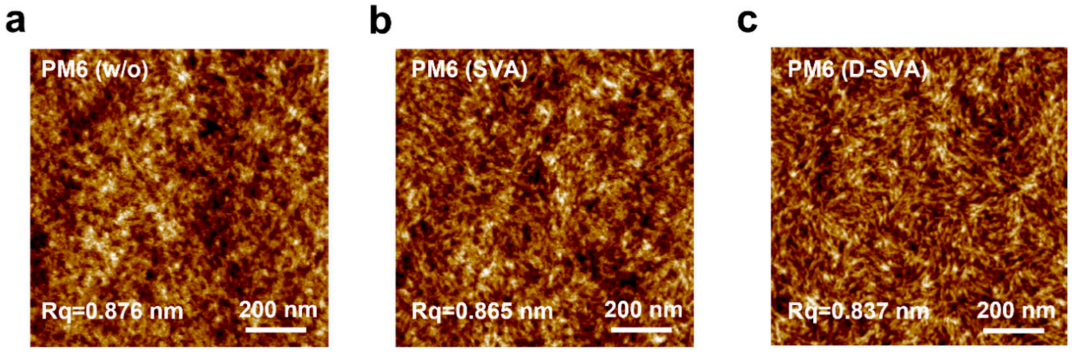

Figure S11. Atomic force microscopy (AFM) images of PM6 donor films. (a) without treatment (w/o), (b) after solvent vapor annealing (SVA), and (c) after dual-step solvent vapor annealing (D-SVA). Scale bar: 200 nm.

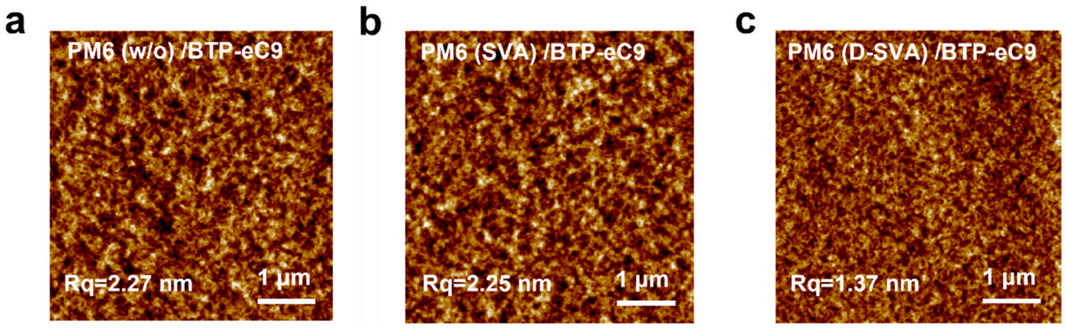

Figure S12. AFM images of bilayer active-layer films based on PM6 films subjected to different treatment methods. (a) without treatment (w/o), (b) solvent vapor annealing (SVA), (c) dual-step solvent vapor annealing (D-SVA).

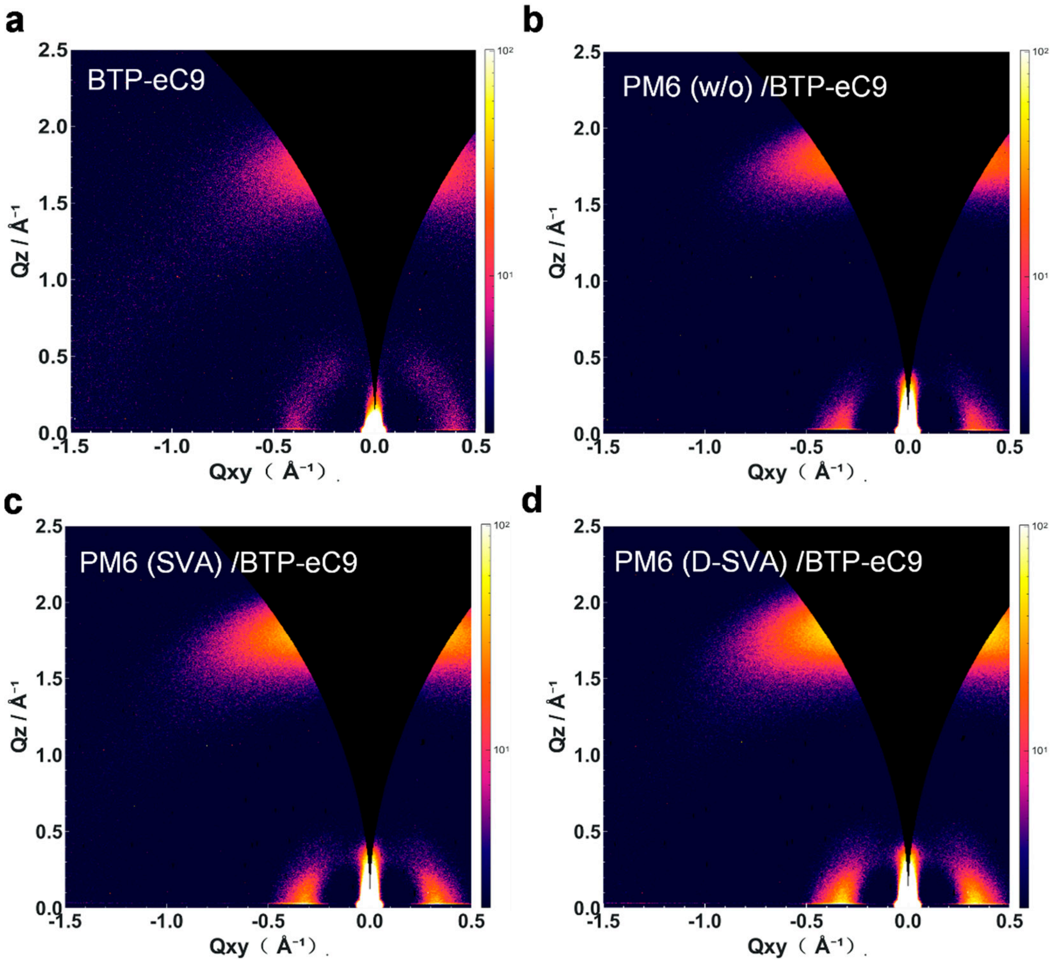

Figure S13. The 2D GIWAXS scattering patterns of different active layers. (a) BTP-eC9, (b) PM6(w/o)/BTP-eC9, (c) PM6(SVA)/BTP-eC9, (d) PM6(D-SVA)/BTP-eC9.

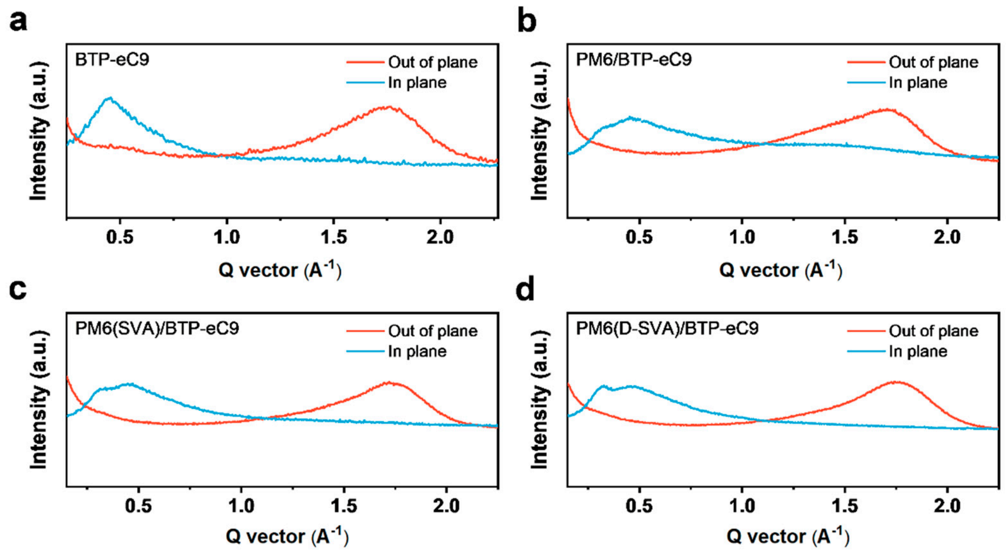

Figure S14. Line scattering profiles cut from the 2D GIWAXS patterns of different active layers in out-of-plane (OOP) and in-plane (IP) directions. (a) BTP-eC9, (b) PM6(w/o)/BTP-eC9, (c) PM6(SVA)/BTP-eC9, (d) PM6(D-SVA)/BTP-eC9.

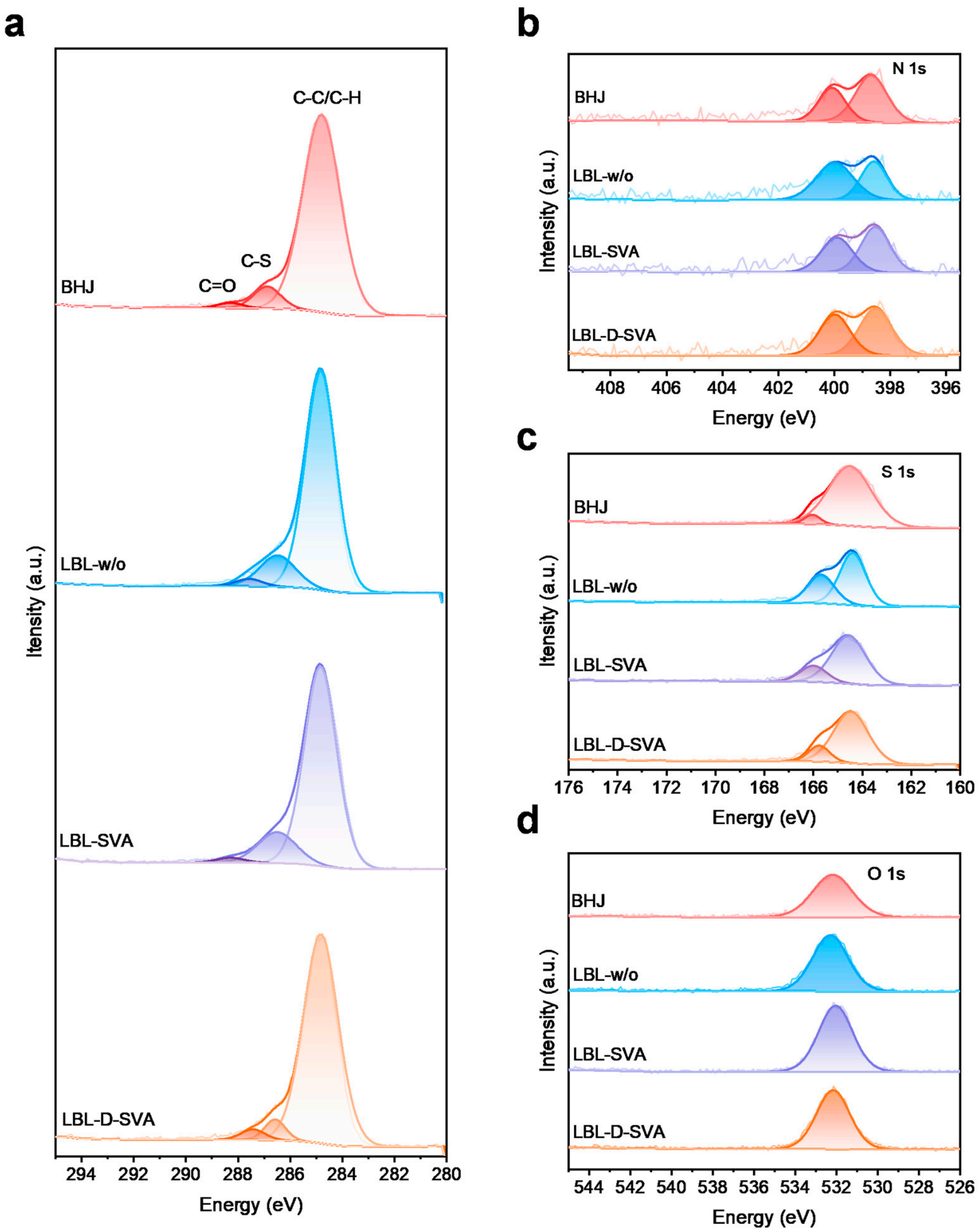

Figure S15. XPS spectra of different elements in active layers. (a) C, (b) N, (c) O, (d) S.

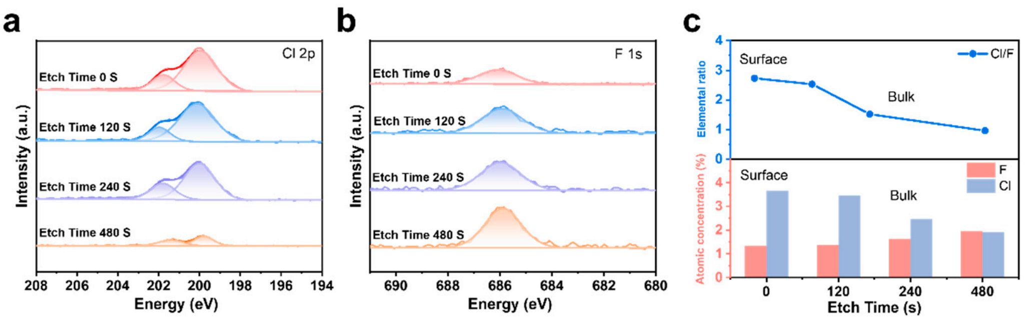

Figure S16. Depth-profiled XPS (DP-XPS) analysis of Cl and F elements in the film. (a) Cl 2p spectra at different etching times, (b) F 1s spectra at different etching times, (c) Cl/F elemental ratio and Cl, F atomic concentration versus etching time.

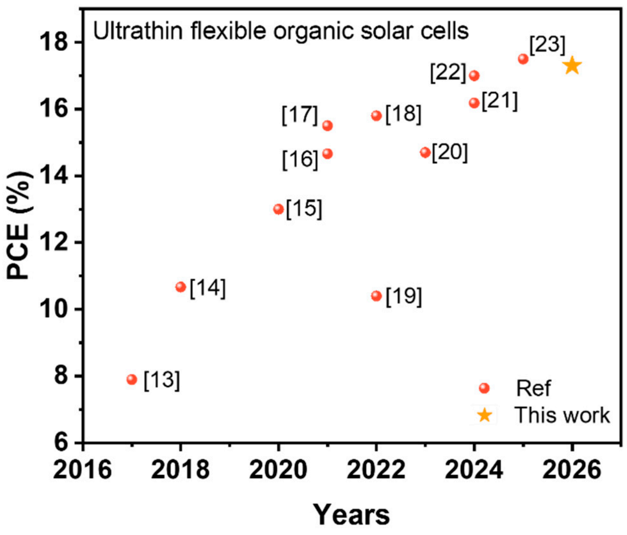

Figure S17. Summary of power conversion efficiencies (PCE) of ultrathin flexible organic solar cells.

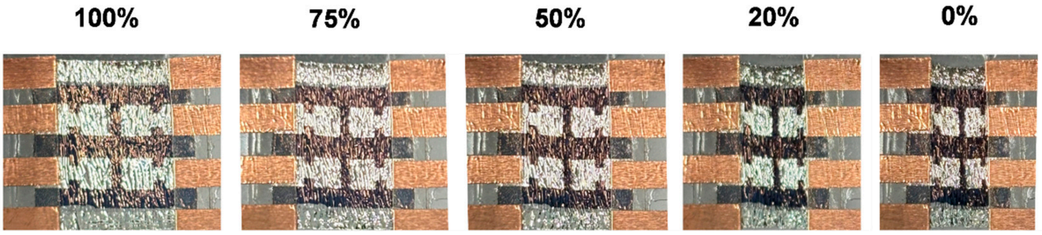

Figure S18. Photographs of ultrathin flexible organic solar cells at different compression levels.

Table S1. Photovoltaic parameters of PM6/BTP-eC9 devices for different optimization times.

CB (min)	CS_2 (s)	V_{OC} (V)	FF (%)	J_{SC} (mA cm^{-2})	J_{SC} ^{calc} (mA cm^{-2}) [a)]	PCE (%) [b)]
0	0	0.846	76.8	27.9	26.91	18.1 (17.8±0.3)
	20	0.847	76.9	28.1	27.15	18.3 (18.0±0.3)
	40	0.848	77.1	28.2	27.22	18.4 (18.2±0.2)
	60	0.846	76.9	27.7	26.88	18.0 (17.8±0.2)
1	0	0.847	76.9	28.1	27.07	18.3 (18.0±0.3)
	20	0.848	77.1	28.4	27.49	18.6 (18.3±0.3)
	40	0.847	77.1	28.2	27.21	18.4 (18.2±0.2)
	60	0.846	76.9	27.9	26.88	18.2 (18.0±0.2)
3	0	0.849	77.6	28.1	27.23	18.5 (18.2±0.3)
	20	0.853	77.9	28.4	27.41	18.9 (18.7±0.2)
	40	0.853	77.7	28.3	27.34	18.7 (18.4±0.3)
	60	0.851	77.0	27.9	27.00	18.3 (18.2±0.1)
5	0	0.849	77.7	28.2	27.09	18.6 (18.3±0.3)
	20	0.853	77.9	28.4	27.30	18.9 (18.7±0.2)
	40	0.857	78.3	28.8	27.69	19.3 (19.1±0.2)
	60	0.851	78.2	28.5	27.40	19.0 (18.5±0.8)

Table S1(continued). Photovoltaic parameters of PM6/BTP-eC9 devices for different optimization times.

CB [a)] (min)	CS_2 (s)	V_{OC} (V)	FF (%)	J_{SC} (mA cm^{-2})	J_{SC}^{calc} (mA cm^{-2}) [a)]	PCE (%) [b)]
7	0	0.852	77.4	28.2	27.21	18.6 (18.4±0.2)
	20	0.854	77.6	28.5	27.58	18.9 (18.7±0.2)
	40	0.856	78.0	28.7	27.76	19.2 (18.9±0.3)
	60	0.851	78.0	28.2	27.14	18.7 (18.6±0.1)
10	0	0.848	76.7	28.0	26.86	18.2 (18.1±0.1)
	20	0.847	76.8	28.2	27.10	18.4 (18.2±0.2)
	40	0.849	77.7	28.5	27.46	18.8 (18.5±0.3)
	60	0.846	76.9	28.2	27.03	18.3 (18.1±0.2)

[a)] Calculated current densities from EQE spectra; [b)] The average results from 20 devices

Table S2. The statistical data of sequentially deposited rigid organic solar cells based on PM6 donor under different strategies

Time	Device structure	Strategy	PCE (%)	Ref
2020.3	ITO/PEDOT:PSS/PM6/Y6:FBR/PFN-Br/Ag	Ternary	16.4	[1]
2020.12	ITO/PEDOT:PSS/PM6/Y6-BO(CN)/PDINN/Ag	Additive	17.2	[2]
2021.1	ITO/PEDOT:PSS/PM6/BO-4Cl:BTP-S2/PFN-Br/Ag	Ternary	18.16	[3]
2022.9	ITO/PEDOT:PSS/PM6:PM7/Y6:O1-2F/PDINN/Ag	Quaternary	18.23	[4]
2023.1	ITO/PEDOT:PSS/PM6/BO-4Cl:L8-BO/PDINN/Ag	Ternary	17.89	[5]
2023.10	ITO/PEDOT:PSS/PM6:BTP-eC9:L8-BO/BTP-eC9:L8-BO/PDINN/Ag	Thermal Annealing	18.3	[6]
2024.1	ITO/PEDOT:PSS/PM6:L8-BO/L8-BO:PM6/PDINN/Ag	Mutually Diluted	19.4	[7]
2024.6	ITO/PEDOT:PSS/PM6/L8-BO/PDINN/Ag	Additive	19.03	[8]
2025.3	ITO/2PACz/PM6(AO1010) BTP-eC9/PDINN/Ag	Additive	18.67	[9]
2025.4	ITO/2PACz/PM6/BTP-eC9(BIP)/ PNDIT-F3N/Ag	Additive	19.63	[10]
2025.6	ITO/PEDOT:PSS/PM6(4Dz)/BTP-eC9/PDINN/Ag	Photocrosslinking	12.6	[11]
2026.4	ITO/PEDOT:PSS/PM6/NPs/BTP-eC9 /PDINN/Ag	Intermediate Layer	17.94	[12]
This work	ITO/Br-2PACz/PM6/BTP-eC9/PDINN/Ag	D-SVA	19.3	

Table S3. Summary of rigid OSCs photovoltaic parameters for different active layer systems.

Device	Condition	V_{OC} (V)	FF (%)	J_{SC} (mA cm^{-2})	$J_{SC}{}^{calc}$ (mA cm^{-2}) [a)]	PCE (%) [b)]
PM6/Y6	w/o	0.838 (0.836±0.002)	75.8 (75.5±0.3)	26.4 (26.1±0.3)	25.4	16.8 (16.6±0.2)
	SVA	0.841 (0.838±0.003)	76.7 (76.5±0.2)	26.7 (26.5±0.2)	25.7	17.2 (17.0±0.2)
	D-SVA	0.843 (0.841±0.002)	77.5 (77.3±0.2)	27.1 (26.9±0.2)	26.0	17.7 (17.6±0.1)
D18/N3	w/o	0.844 (0.841±0.003)	76.0 (75.6±0.4)	27.3 (27.0±0.3)	26.3	17.5 (17.3±0.2)
	SVA	0.847 (0.845±0.002)	76.8 (76.5±0.3)	27.7 (27.5±0.2)	26.7	18.1 (17.9±0.2)
	D-SVA	0.853 (0.851±0.002)	77.7 (77.5±0.2)	28.0 (27.8±0.2)	26.8	18.5 (18.3±0.2)
PM6/PY-IT	w/o	0.931 (0.928±0.003)	74.1 (73.8±0.3)	24.0 (23.7±0.3)	23.2	16.6 (16.4±0.2)
	SVA	0.944 (0.942±0.002)	74.5 (74.2±0.3)	24.4 (24.1±0.3)	23.6	17.1 (16.9±0.2)
	D-SVA	0.951 (0.949±0.002)	74.8 (74.6±0.2)	24.8 (24.7±0.1)	24.0	17.6 (17.5±0.1)

[a)] Calculated current densities from EQE spectra; [b)] The average results from 20 devices

Table S4. J_{ph}, J_{sat}, and J_{ph}/J_{sat} values of different processed devices.

Treatment	J_{sat} (mA/cm^2)	J_{ph}^* (mA/cm^2)	$J_{ph}^\#$ (mA/cm^2)	η_{diss} (%)	η_{coll} (%)
w/o	28.83	27.93	25.07	96.9	86.9
SVA	29.11	28.40	25.52	97.6	87.6
D-SVA	29.32	28.79	25.97	98.2	88.5

J_{ph}^* - photocurrent density in short circuit state

$J_{ph}^\#$ - the photocurrent density at the maximum output power state

Table S5. Electron and hole mobilities and mobility ratios for different treatments.

Condition	Hole mobility (μ_h) (cm^2 V^{-1} s^{-1})	Electron mobility (μ_e) (cm^2 V^{-1} s^{-1})	μ_h/μ_e
w/o	3.63×10^{-4}	2.17×10^{-4}	1.67
SVA	4.14×10^{-4}	3.31×10^{-4}	1.25
D-SVA	4.53×10^{-4}	4.11×10^{-4}	1.10

Table S6. Analytical data of GIWAXS results for PM6 films under different treatment methods.

	Samples	Peak	q (Å^{-1})	d-spacing (Å)	FWHMW (Å^{-1})	CCL (Å)
(IP)	w/o	Lamellar	0.299	21.01	0.084	67.3
		π-π				
	SVA	Lamellar	0.303	20.74	0.081	69.8
		π-π				
	D-SVA	Lamellar	0.312	20.14	0.07	73.4
		π-π				
(OOP)	w/o	Lamellar	0.327	19.21	0.106	53.3
		π-π	1.69	3.72	0.517	10.9
	SVA	Lamellar	0.332	18.93	0.101	56.0
		π-π	1.70	3.70	0.482	11.7
	D-SVA	Lamellar	0.341	18.43	0.096	58.9
		π-π	1.71	3.67	0.464	12.2

Table S7. Analytical data of GIWAXS results for BTP-eC9 films.

Samples	Peak	q ($Å^{-1}$)	d-spacing (Å)	FWHMW ($Å^{-1}$)	CCL (Å)
(IP)	Lamellar	0.456	13.78	0.327	17.3
	π-π				
(OOP)	Lamellar				
	π-π	1.760	3.57	0.441	12.8

Table S8. Analytical data of GIWAXS results for bilayer active-layer films under different treatment methods.

	Samples	Peak	q ($Å^{-1}$)	d-spacing (Å)	FWHMW ($Å^{-1}$)	CCL (Å)
(IP)	w/o	Lamellar	0.465	13.87	0.231	24.5
			0.336	18.70	0.273	20.7
		π-π				
	SVA	Lamellar	0.448	14.02	0.217	26.1
			0.315	19.95	0.262	21.6
		π-π				
	D-SVA	Lamellar	0.465	13.51	0.201	28.1
			0.316	19.88	0.248	22.8
		π-π				
(OOP)	w/o	Lamellar				
		π-π	1.713	3.67	0.459	12.3
	SVA	Lamellar				
		π-π	1.735	3.62	0.426	13.3
	D-SVA	Lamellar				
		π-π	1.765	3.56	0.399	14.2

Table S9. Atomic concentrations of active layers obtained from XPS under different treatment methods.

Conditions	C (%)	S (%)	O (%)	F (%)	N (%)	Cl (%)	Total (%)
BHJ	76.62	5.94	9.3	1.84	4.29	2.02	100
LBL-w/o	74.93	5.21	11.29	1.78	4.57	2.23	100
LBL-SVA	74.91	5.06	11.26	1.55	4.76	2.46	100
LBL-D-SVA	74.95	4.76	12.07	1.65	4.31	2.25	100

Table S10. Summary of ultrathin flexible OSCs photovoltaic parameters based on different treatments.

Condition [a)]	V_{OC} (V)	FF (%)	J_{SC} (mA cm^{-2})	J_{SC}^{calc} (mA cm^{-2}) [b)]	PCE (%)
w/o	0.846<br>(0.843±0.003)	74.9<br>(74.6±0.3)	25.7<br>(25.5±0.2)	24.6	16.3<br>(16.0±0.3)
SVA	0.847<br>(0.844±0.003)	75.4<br>(75.2±0.2)	26.1<br>(25.8±0.3)	25.2	16.7<br>(16.5±0.2)
D-SVA	0.852<br>(0.849±0.003)	75.9<br>(75.7±0.2)	26.7<br>(26.5±0.2)	25.7	17.3<br>(17.1±0.2)

[a)] The average results from 20 devices; [b)] Calculated current densities from EQE spectra.

Table S11. The statistical data for ultrathin flexible organic solar cells.

Time	Device structure	PCE (%)	Ref
2017.9	Parylene/ITO/ZnO/PNTz4T:PC71BM/MoO_3/Ag	7.9	[13]
2018.9	Parylene/ITO/ZnO/PBDTTT OFT:PC71BM/MoO_3/Ag	10.67	[14]
2020.1	Parylene/SU8/ITO/ZnO/PBDTTT-OFT:IEICO 4F:$PC_{71}BM$/MoO_3/Ag	13.0	[15]
2021.3	PE/PH1000/ PEDOT:PSS /PM6:Y6/PFN-Br/Al	14.66	[16]
2021.7	PET/PH1000/PEDOT:PSS/D18-Cl:Y6:$PC_{71}BM$/PFNDI-Br/Ag	15.5	[17]
2022.3	tPI/ITO/PEI-Zn/PM6:Y6/MoO_3/Ag	15.8	[18]
2022.6	Parylene/ITO/ZnO/PBDTTT-OFT:IEICO-4F/PEDOT:PSS/Ag	10.4	[19]
2023.3	tPI/ITO/PEI-Zn/PM6:Y6/MoO_3/Ag	14.7	[20]
2024.8	Parylene/ITO/ZnO/PM6:O-IDTBR:Y6/MoO_x/Ag	16.18	[21]
2024.11	Parylene/ITO/IZO/PM6:L8-BO:BTP-eC9/MoO_3/Ag	17.0	[22]
2025.8	Parylene/ITO/m-SAM/PM6:L8-BO:BTP-eC9/PDINN/Ag	17.5	[23]
This work	Parylene/ITO/Br-2PACz/PM6/BTP-eC9/PDINN/Ag	17.3	

Reference

1. Ren, M.; Zhang, G.; Chen, Z.; Xiao, J.; Jiao, X.; Zou, Y.; Yip, H.-L.; Cao, Y. High-Performance Ternary Organic Solar Cells with Controllable Morphology via Sequential Layer-by-Layer Deposition. *ACS Appl. Mater. Interfaces* **2020**, *12*, 13077–13086.
2. Fu, H.; Gao, W.; Li, Y.; Lin, F.; Wu, X.; Son, J.H.; Luo, J.; Woo, H.Y.; Zhu, Z.; Jen, A.K. -Y. A Generally Applicable Approach Using Sequential Deposition to Enable Highly Efficient Organic Solar Cells. *Small Methods* **2020**, *4*, 2000687.
3. Zhan, L.; Li, S.; Xia, X.; Li, Y.; Lu, X.; Zuo, L.; Shi, M.; Chen, H. Layer-by-Layer Processed Ternary Organic Photovoltaics with Efficiency over 18%. *Adv. Mater.* **2021**, *33*, 2007231.
4. Li, S.; Jia, Z.; Ma, Q.; Wu, Y.; Meng, Q.; Zhang, J.; Qiu, B.; Qiao, J.; Li, Y. Highly Efficient Layer-by-Layer Processed Quaternary Organic Solar Cells with Improved Charge Transport and Reduced Energy Loss. *Solar RRL* **2022**, *6*, 2200496.
5. Li, X.; Yang, H.; Du, X.; Lin, H.; Yang, G.; Zheng, C.; Tao, S. High-Performance Layer-by-Layer Organic Solar Cells Enabled by Non-Halogenated Solvent with 17.89% Efficiency. *Chem. Eng. J.* **2023**, *452*, 139496.
6. Li, S.; Shi, C.; Gong, Y.; Yang, K.; Luo, X.; Jiang, L.; Zhu, H.; Lu, X.; Yuan, J.; Zou, Y. Additive-Free All-Green Solvent-Processed Efficient and Stable Pseudo-Bilayer Bulk Heterojunction Ternary Organic Solar Cells. *J. Phys. Chem. C* **2023**, *127*, 19918–19926.
7. Wang, L.; Chen, C.; Fu, Y.; Guo, C.; Li, D.; Cheng, J.; Sun, W.; Gan, Z.; Sun, Y.; Zhou, B.; et al. Donor–Acceptor Mutually Diluted Heterojunctions for Layer-by-Layer Fabrication of High-Performance Organic Solar Cells. *Nat. Energy* **2024**, *9*, 208–218.
8. Wu, W.; Luo, Y.; Dela Peña, T.A.; Yao, J.; Qammar, M.; Li, M.; Yan, H.; Wu, J.; Ma, R.; Li, G. Defining Solid Additive's Pivotal Role on Morphology Regulation in Organic Solar Cells Produced by Layer-by-layer Deposition. *Adv. Energy Mater.* **2024**, *14*, 2400354.
9. Wang, Q.; Zhao, S.; Ding, H.; Zhu, P.; Fu, Y.; Lv, Y.; Lu, X.; Zhu, H.; Liao, X.; Chen, Y. Enhancing Stability and Performance of Pseudo-Planar Heterojunction Organic Solar Cells Using a Hindered Phenolic Antioxidant with over 19% Efficiency. *Sci. China Mater.* **2025**, *68*, 838–849.
10. Li, X.; Du, X.; Zhao, J.; Lin, H.; Zheng, C.; Tao, S. Layer-by-Layer Solution Processing Method for Organic Solar Cells. *Solar RRL* **2021**, *5*, 2000592.
11. Suzuki, R.; Nakano, K.; Miyasaka, M.; Tajima, K. Vertical Component Distributions in Organic Solar Cells Controlled by Photocrosslinking and Layer-by-Layer Deposition. *Small* **2025**, *21*, 2411988.
12. Sun, L.; Zhang, X.; Wang, X.; Gao, C.; Yang, R.; Zhang, G.; Zou, X.; Xu, H.; Kan, Y.; Sun, Y.; et al. Aqueous Organic Nanoparticle-Mediated Precise Modulation of -Donor-Acceptor Interfaces Enhances Exciton Dissociation towards 20.6 % Efficiency in Green Solvent-Processed Organic Solar Cells. *Mater. Sci. Eng. R* **2026**, *169*, 101201.
13. Jinno, H.; Fukuda, K.; Xu, X.; Park, S.; Suzuki, Y.; Koizumi, M.; Yokota, T.; Osaka, I.; Takimiya, K.; Someya, T. Stretchable and Waterproof Elastomer-Coated Organic Photovoltaics for Washable Electronic Textile Applications. *Nat. Energy* **2017**, *2*, 780–785.
14. Park, S.; Heo, S.W.; Lee, W.; Inoue, D.; Jiang, Z.; Yu, K.; Jinno, H.; Hashizume, D.; Sekino, M.; Yokota, T.; et al. Self-Powered Ultra-Flexible Electronics via Nano-Grating-Patterned Organic Photovoltaics. *Nature* **2018**, *561*, 516–521.
15. Huang, W.; Jiang, Z.; Fukuda, K.; Jiao, X.; McNeill, C.R.; Yokota, T.; Someya, T. Efficient and Mechanically Robust Ultraflexible Organic Solar Cells Based on Mixed Acceptors. *Joule* **2020**, *4*, 128–141.
16. Wan, J.; Wen, R.; Xia, Y.; Dai, M.; Huang, H.; Xue, L.; Zhang, Z.; Fang, J.; Hui, K.N.; Fan, X. All Annealing-Free Solution-Processed Highly Flexible Organic Solar Cells. *J. Mater. Chem. A* **2021**, *9*, 5425–5433.

17. Song, W.; Yu, K.; Zhou, E.; Xie, L.; Hong, L.; Ge, J.; Zhang, J.; Zhang, X.; Peng, R.; Ge, Z. Crumple Durable Ultraflexible Organic Solar Cells with an Excellent Power-per-Weight Performance. *Adv. Funct. Mater.* **2021**, *31*, 2102694.
18. Xiong, S.; Fukuda, K.; Lee, S.; Nakano, K.; Dong, X.; Yokota, T.; Tajima, K.; Zhou, Y.; Someya, T. Ultrathin and Efficient Organic Photovoltaics with Enhanced Air Stability by Suppression of Zinc Element Diffusion. *Adv. Sci.* **2022**, *9*, 2105288.
19. Rich, S.I.; Lee, S.; Fukuda, K.; Someya, T. Developing the Nondevelopable: Creating Curved-Surface Electronics from Nonstretchable Devices. *Adv. Mater.* **2022**, *34*, 2106683.
20. Du, B.; Fukuda, K.; Yokota, T.; Inoue, D.; Hashizume, D.; Xiong, S.; Lee, S.; Takakuwa, M.; Sun, L.; Wang, J.; et al. Surface-Energy-Mediated Interfacial Adhesion for Mechanically Robust Ultraflexible Organic Photovoltaics. *ACS Appl. Mater. Interfaces* **2023**, *15*, 14624–14633.
21. Saifi, S.; Xiao, X.; Cheng, S.; Guo, H.; Zhang, J.; Müller-Buschbaum, P.; Zhou, G.; Xu, X.; Cheng, H.-M. An Ultraflexible Energy Harvesting-Storage System for Wearable Applications. *Nat. Commun* **2024**, *15*, 6546.
22. Liu, X.; Ji, Y.; Xia, Z.; Zhang, D.; Cheng, Y.; Liu, X.; Ren, X.; Liu, X.; Huang, H.; Zhu, Y.; et al. In-Doped ZnO Electron Transport Layer for High-Efficiency Ultrathin Flexible Organic Solar Cells. *Adv. Sci.* **2024**, *11*, 2402158.
23. Ji, Y.; Liang, L.; Chen, T.; Liu, X.; Mao, M.; Hu, Y.; Jin, Y.; Huang, X.; Ren, X.; Xie, D.; et al. High-Performance Ultrathin Flexible Organic Solar Cells Through the Modification of Self-Assembled Monolayers. *Adv. Energy Mater.* **2025**, *15*, 2501698.
